# EBD: an eye biomarker database

**DOI:** 10.1093/bioinformatics/btad194

**Published:** 2023-04-13

**Authors:** Xueli Zhang, Lingcong Kong, Shunming Liu, Xiayin Zhang, Xianwen Shang, Zhuoting Zhu, Yu Huang, Shuo Ma, Ha Jason, Katerina V Kiburg, Chunwen Zheng, Yunyan Hu, Cong Li, Guanrong Wu, Yingying Liang, Mengxia He, Yan Wang, Xiaohe Bai, Danli Shi, Wei Wang, Chi Zhang, Ke Zhao, Haining Yuan, Guang Hu, Yijun Hu, Huiying Liang, Honghua Yu, Lei Zhang, Mingguang He

**Affiliations:** Department of Ophthalmology, Guangdong Eye Institute, Guangdong Provincial People’s Hospital (Guangdong Academy of Medical Sciences), Southern Medical University, No. 106 Zhongshan Second Road, Guangzhou 510080, China; Medical Research Institute, Guangdong Provincial People's Hospital (Guangdong Academy of Medical Sciences), Southern Medical University, No. 106 Zhongshan Second Road, Guangzhou 510080, China; Medical Big Data Center, Guangdong Provincial People's Hospital (Guangdong Academy of Medical Sciences), Southern Medical University, No. 106 Zhongshan Second Road, Guangzhou 510080, China; Department of Ophthalmology, Guangdong Eye Institute, Guangdong Provincial People’s Hospital (Guangdong Academy of Medical Sciences), Southern Medical University, No. 106 Zhongshan Second Road, Guangzhou 510080, China; Department of Ophthalmology, Guangdong Eye Institute, Guangdong Provincial People’s Hospital (Guangdong Academy of Medical Sciences), Southern Medical University, No. 106 Zhongshan Second Road, Guangzhou 510080, China; Department of Ophthalmology, Guangdong Eye Institute, Guangdong Provincial People’s Hospital (Guangdong Academy of Medical Sciences), Southern Medical University, No. 106 Zhongshan Second Road, Guangzhou 510080, China; Department of Ophthalmology, Guangdong Eye Institute, Guangdong Provincial People’s Hospital (Guangdong Academy of Medical Sciences), Southern Medical University, No. 106 Zhongshan Second Road, Guangzhou 510080, China; Department of Ophthalmology, Guangdong Eye Institute, Guangdong Provincial People’s Hospital (Guangdong Academy of Medical Sciences), Southern Medical University, No. 106 Zhongshan Second Road, Guangzhou 510080, China; Medical Big Data Center, Guangdong Provincial People's Hospital (Guangdong Academy of Medical Sciences), Southern Medical University, No. 106 Zhongshan Second Road, Guangzhou 510080, China; Centre for Eye Research Australia, Royal Victorian Eye and Ear Hospital, 32 Gisborne Street, East Melbourne, VIC 3002, Australia; Centre for Eye Research Australia, Royal Victorian Eye and Ear Hospital, 32 Gisborne Street, East Melbourne, VIC 3002, Australia; Department of Ophthalmology, Guangdong Eye Institute, Guangdong Provincial People’s Hospital (Guangdong Academy of Medical Sciences), Southern Medical University, No. 106 Zhongshan Second Road, Guangzhou 510080, China; Department of Ophthalmology, Guangdong Eye Institute, Guangdong Provincial People’s Hospital (Guangdong Academy of Medical Sciences), Southern Medical University, No. 106 Zhongshan Second Road, Guangzhou 510080, China; Department of Ophthalmology, Guangdong Eye Institute, Guangdong Provincial People’s Hospital (Guangdong Academy of Medical Sciences), Southern Medical University, No. 106 Zhongshan Second Road, Guangzhou 510080, China; Department of Ophthalmology, Guangdong Eye Institute, Guangdong Provincial People’s Hospital (Guangdong Academy of Medical Sciences), Southern Medical University, No. 106 Zhongshan Second Road, Guangzhou 510080, China; Department of Ophthalmology, Guangdong Eye Institute, Guangdong Provincial People’s Hospital (Guangdong Academy of Medical Sciences), Southern Medical University, No. 106 Zhongshan Second Road, Guangzhou 510080, China; Department of Ophthalmology, Guangdong Eye Institute, Guangdong Provincial People’s Hospital (Guangdong Academy of Medical Sciences), Southern Medical University, No. 106 Zhongshan Second Road, Guangzhou 510080, China; Department of Ophthalmology, Guangdong Eye Institute, Guangdong Provincial People’s Hospital (Guangdong Academy of Medical Sciences), Southern Medical University, No. 106 Zhongshan Second Road, Guangzhou 510080, China; Department of Mathematics, University of California, 9500 Gilman Dr, La Jolla, San Diego, CA 92093, United States; State Key Laboratory of Ophthalmology, Zhongshan Ophthalmic Center, Sun Yat-sen University, No. 54 Xianlie South Road, Guangzhou 510060, China; State Key Laboratory of Ophthalmology, Zhongshan Ophthalmic Center, Sun Yat-sen University, No. 54 Xianlie South Road, Guangzhou 510060, China; Department of Otolaryngology, Guangzhou Women and Children's Medical Centre, No. 9 Jinsui Road, Guangzhou 510623, China; Department of Radiology, Guangdong Provincial People's Hospital (Guangdong Academy of Medical Sciences), Southern Medical University, No. 106 Zhongshan Second Road, Guangzhou 510080, China; HangZhou Medical College, No. 8 Jikang Street, Lin'an District, Hangzhou 311399, China; Department of Bioinformatics, Center for Systems Biology, School of Biology and Basic Medical Sciences, Soochow University, No. 199 Ren'ai Road, Suzhou Industrial Park, Suzhou 215123, China; Department of Ophthalmology, Guangdong Eye Institute, Guangdong Provincial People’s Hospital (Guangdong Academy of Medical Sciences), Southern Medical University, No. 106 Zhongshan Second Road, Guangzhou 510080, China; Medical Big Data Center, Guangdong Provincial People's Hospital (Guangdong Academy of Medical Sciences), Southern Medical University, No. 106 Zhongshan Second Road, Guangzhou 510080, China; Department of Ophthalmology, Guangdong Eye Institute, Guangdong Provincial People’s Hospital (Guangdong Academy of Medical Sciences), Southern Medical University, No. 106 Zhongshan Second Road, Guangzhou 510080, China; Centre for Eye Research Australia, Royal Victorian Eye and Ear Hospital, 32 Gisborne Street, East Melbourne, VIC 3002, Australia; China-Australia Joint Research Center for Infectious Diseases, School of Public Health, Xi'an Jiaotong University Health Science Center, No.76 Yanta West Road, Xi'an 710061, China; Artificial Intelligence and Modelling in Epidemiology Program, Melbourne Sexual Health Centre, Alfred Health, 55 Commercial Rd, Melbourne, VIC 3004, Australia; Central Clinical School, Faculty of Medicine, Monash University, 27 Rainforest Walk, Melbourne, Clayton VIC 3800, Australia; Department of Ophthalmology, Guangdong Eye Institute, Guangdong Provincial People’s Hospital (Guangdong Academy of Medical Sciences), Southern Medical University, No. 106 Zhongshan Second Road, Guangzhou 510080, China; Centre for Eye Research Australia, Royal Victorian Eye and Ear Hospital, 32 Gisborne Street, East Melbourne, VIC 3002, Australia; State Key Laboratory of Ophthalmology, Zhongshan Ophthalmic Center, Sun Yat-sen University, No. 54 Xianlie South Road, Guangzhou 510060, China

## Abstract

**Motivation:**

Many ophthalmic disease biomarkers have been identified through comprehensive multiomics profiling, and hold significant potential in advancing the diagnosis, prognosis, and management of diseases. Meanwhile, the eye itself serves as a natural biomarker for several systemic diseases including neurological, renal, and cardiovascular systems. We aimed to collect and standardize this eye biomarkers information and construct the eye biomarker database (EBD) to provide ophthalmologists with a platform to search, analyze, and download these eye biomarker data.

**Results:**

In this study, we present the EBD <http://www.eyeseeworld.com/ebd/index.html>, a world-first online compilation comprising 889 biomarkers for 26 ocular diseases and 939 eye biomarkers for 181 systemic diseases. The EBD also includes the information of 78 “nonbiomarkers”—the objects that have been proven cannot be biomarkers. Biological function and network analysis were conducted for these ocular disease biomarkers, and several hub pathways and common network topology characteristics were newly identified, which may promote future ocular disease biomarker discovery and characterizes the landscape of biomarkers for eye diseases at the pathway and network level. The EBD is expected to yield broader utility among developmental biologists and clinical scientists in and outside of the eye field by assisting in the identification of biomarkers linked to eye disorders and related systemic diseases.

**Availability and implementation:**

EBD is available at http://www.eyeseeworld.com/ebd/index.html.

## 1 Introduction

At least 2.2 billion people worldwide suffer from visual impairment or blindness, at least half of which are preventable or curable (World Health Organization 2019). This major disease burden significantly impacts individuals, and greatly increases the medical, social, and socioeconomic burden of disease. Meanwhile, the incidence of major eye diseases such as refractive errors (RE; mainly including myopia, hyperopia, presbyopia, and astigmatism), cataract, diabetic retinopathy (DR), age-related macular degeneration (AMD), and glaucoma continues to rise (Strimbu and Tavel 2010; Tham et al. 2014; Wong et al. 2014b; Flaxman et al. 2017).

The advent of precision and network medicine in recent years has sparked a great interest in the role of disease biomarkers, which may improve diagnosis and therapy of complex diseases (Strimbu and Tavel 2010). Analysis of biomarkers across different tissues, and longitudinal monitoring allow researchers to characterize the genetic controls of eye development and function (Tamhane et al. 2019). However, the clinical utility of comprehensive biological tests and related pathways for ocular disease and systemic diseases with ocular manifestations remains largely unexplored (Tamhane et al. 2019). Furthermore, an improved understanding of protein–protein interactions (PPIs) and their collective function remains a key priority and network analysis provides a powerful tool for investigating protein regulation and explaining their integrated biological function (Barabási 2009; De las Rivas and Fontanillo 2012).

As the majority of ocular diseases could be avoided with early diagnosis and intervention, an improved system for biomarker discovery and linkage is required (Barabási 2009). As of May 2022, more than 14 000 papers have been published on eye biomarkers; however, its discovery potential is greatly diminished by decentralized data collection, and the lack of standardization of the data. Hence, there is an urgent need for a platform that covers all identified ocular disease biomarkers with curated biomedicine information, and interaction networks. This allows for further understanding of the network of biomarker functions and interactions. In addition, the eye itself serves as an important biomarker, and a window into the function and health of various body systems, in both physiological and pathological states (Wong et al. 2005, 2014a; London et al. 2013). Further studies have focused on the eye as a biomarker for systemic diseases (Vujosevic et al. 2023; Zhu et al. 2023).

In other fields, several biomarker databases for human complex diseases: CBD for colorectal cancers (Zhang et al. 2018), HFBD for Heart failure (He et al. 2021), and IDBD for infectious diseases (Yang et al. 2008). These biomarker datasets have offered great help to researchers. In the ophthalmology field, Yuan et al. (2021) have presented the EyeDiseases database, which collected information on eye diseases from multiomics data. However, the genes contained in the EyeDiseases database only showed statistical associations with eye diseases, but have not been verified as potential clinical biomarkers for eye diseases. Wolf et al. reported the Human Eye Transcriptome Atlas (Wolf et al. 2022), which covered the web-based transcriptome data for 100 diseased and healthy human eye specimens. However, the Human Eye Transcriptome Atlas only contained eye disease-related data, not experimentally confirmed biomarkers. Thus, a platform that covers standard and ontology records of the eye as biomarkers of systemic diseases remains an urgent demand.

In this article, we presented a human EBD <http://www.eyeseeworld.com/ebd/index.html>, a comprehensive platform for human eye biomarkers, which was manually curated and integrated different annotations: genes, proteins, metabolites, networks, pathways, diseases, images, and machine indexes, to fill these gaps. EBD encompassed of 889 biomarkers for 26 eye diseases and 939 eye biomarkers for 181 systemic diseases, included nucleic acids-based, protein, metabolite, and some specific biomarkers, such as image biomarkers and nonbiomarkers, which could help researchers avoid previous mistakes and improve the precision of biomarker discovery (Supplementary Fig. S1). The database provided expression information, biomarker–biomarker interaction (BBI) networks, pathway enrichment, and network function information for biomarkers.

In summary, the conception of EBD provided a standardized platform for ocular biomarkers, and might be a future driver for ophthalmic precision medicine. This user-friendly database facilitates the search, analysis, and download of standard eye biomarker information, and characterizes a landscape for ocular biomarkers at pathway and network levels, and provides biological insights through genomic, transcriptomic, epigenomic, proteomic, and phenomics profiling from ocular diseases and related systemic diseases.

## 2 Materials and methods

### 2.1 Data collection and curation

The literature search was conducted on PubMed, until October 2021. We found 17 637 papers concerning ocular disease biomarkers and 14 804 papers about the eye as the biomarker for body conditions/diseases. The list of these papers was presented in the Download page of the EBD database.

We selected papers that satisfied the following criteria:

1. The studies explicitly state that the subjects studied could be used as any biomarkers for human ocular diseases or eye biomarkers for systemic diseases.
2. The studies conduct the experiment with a control group and demographics characteristics to validate its conclusion.
3. Detailed experimental design and methods were described clearly in the paper.
4. Prediction/diagnosis biomarkers had a sensitivity/specificity/area under the curve >0.7; and the *P*-value of odds ratio/hazard ratio/relative risk for treatment/prognosis biomarkers were lower than 0.01.
5. The sample size included in the study should be bigger than 30. The distribution of the number of patients has been plotted in Supplementary Fig. S2.

A quality assessment is conducted to the selected papers. The Critical Appraisal Skills Program (CASP) checklists were used to calculate the confidence score of included papers (https://casp-uk.net/casp-tools-checklists/). The CASP checklists ask 11 each to identify the confidence of the design, methodology, and results of the papers. For most of the questions, users answer “Yes,” “No,” or “Can’t tell” according to the quality of the paper. A paper with ≥ nine “Yes” answers would be considered a good-quality paper. If the number of “Yes” answers ranked between six and eight, the paper would be judged as a normal-quality paper. A paper with less than six “Yes” answers would be categorized as a low-quality paper. We excluded the papers that did not match five or fewer GASP questions. The number of “Yes” answers for each paper had been displayed on the revised webpage. The results of confidence scores were presented in the “Eye diseases” page, “Systemic diseases” page, and “Download” page of the EBD database and Supplementary Tables S1–S3. The distribution of answers for CASP checklists has been presented in Supplementary Fig. S3, which showed that most of the included papers had high quality.

From selected papers, we extracted biomarker information (including biomarker name, biological category, and description) and experiment information (including detailed experimental information: region, race, number, gender, age, source, pivotal method and statistics, application, conclusion, and paper information [confidence score (score in CASP checklists), first author, journal, the impact factor (IF) of the journal (2023), published year, PubMed ID, and the number of citations]).

We used the NCBI Gene database (https://www.ncbi.nlm.nih.gov/gene) and Protein database (https://www.ncbi.nlm.nih.gov/protein/) to annotate standard names and brief descriptions for each biomarker. Gene ontology (GO) information was provided by the UniProt database (https://beta.uniprot.org/). PPI network information for protein biomarkers was collected from the String database (https://string-db.org/). Pathway information for biomarkers was collected from the KEGG database (https://www.kegg.jp/) and Reactome database (https://reactome.org/).

### 2.2 Data analysis

We extracted the protein and gene biomarkers for five major eye diseases: AMD, cataract, DR, glaucoma, and RE, then mapped them separately on the human PPI network to construct the disease-biomarker-specific networks. The source of the PPI information was limited to experiments and databases, and the edge score was set as ≥0.4. Two topology features were used to describe the connectivity of networks: average degree and density. The average degree is used to measure the number of edges compared with the number of nodes in the network, and the higher average degree represents higher connectivity. The density represents the ratio between the edges in a network and the maximum number of edges that the network can include, and the high density indicates high connectivity. For each BBI network, we randomly drew the same size of networks from the human PPI network downloaded from the String database (https://stringdb-static.org/download/protein.info.v11.5.txt.gz), to observe if the nodes in BBI networks were more than expected by chance.

Pathway enrichment analysis and GO annotation were conducted to find common pathways for biomarkers. We summarized the pathways enriched by biomarkers into different diseases, to observe the effect of randomizing biomarkers across disease classes on annotations. The distribution of enriched pathways in different diseases was also calculated. The bootstrap method was used to measure if the links between a disease and a pathway survive randomization.

The expression of biomarkers was also observed on bulk/single-cell RNA-seq (scRNA-seq) data.

### 2.3 Tools and software

The EBD is a MySQL-Apache-based database, and its web interface was built with HTML, PHP, and JavaScript. The String database was used to conduct the PPI network analysis and biological functional analysis (https://string-db.org/). The GTEx database was used to run the expression analysis (https://gtexportal.org/home/).

## 3 Results

### 3.1 Framework of EBD

**Figure 1:**
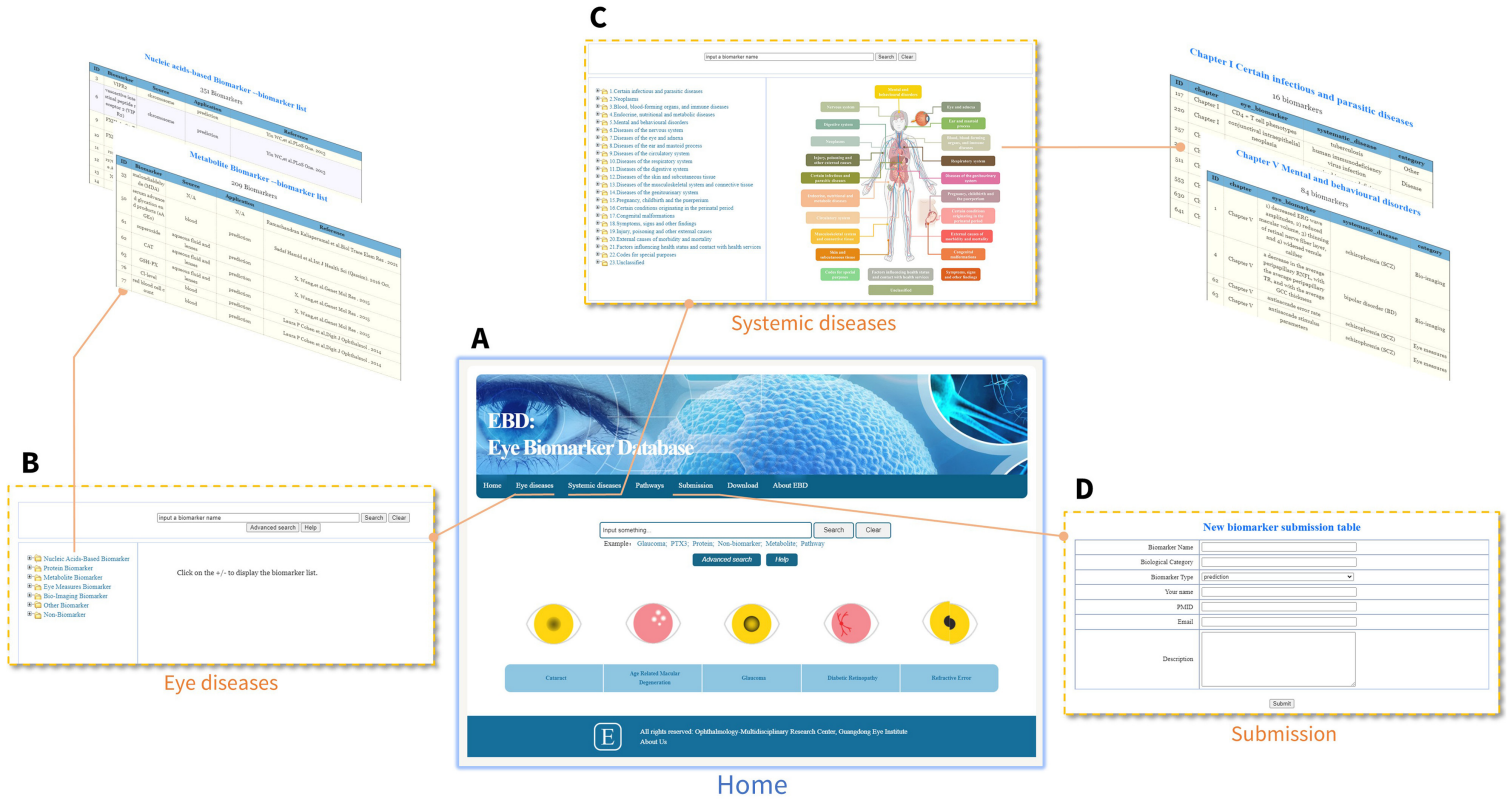
Framework of the EBD. (A) Homepage: includes quick search function for keyword and folder. (B) Eye diseases page: users can search biomedicine information of biomarkers for eye disease via this page. (C) Systemic diseases page: users can search the information of the eye as biomarker for systemic diseases. (D) Submission page: users can submit the new reported biomarkers to us.

The EBD provides a user-friendly interface, which contains seven parts:

1. “Home” page for quick search (Fig. 1A);
2. “Eye diseases” page for the search of biomarkers for eye diseases: biomarker search can be conducted via list search by diseases or biological categories, keyword search, and advanced search (Fig. 1B);
3. “Systemic diseases” page for the searching of the eye as biomarkers for body conditions/diseases (Fig. 1C);
4. “Pathways” page for the search of identified biological pathways for eye diseases biomarkers;
5. Users can submit their newly discovered biomarkers to us via “Submission” page (Fig. 1D);
6. Users can download all the data from “Download” page;
7. “About EBD” page provides basic statistics and analysis results for EBD.

### 3.2 Biological category for biomarkers

**Figure 2:**
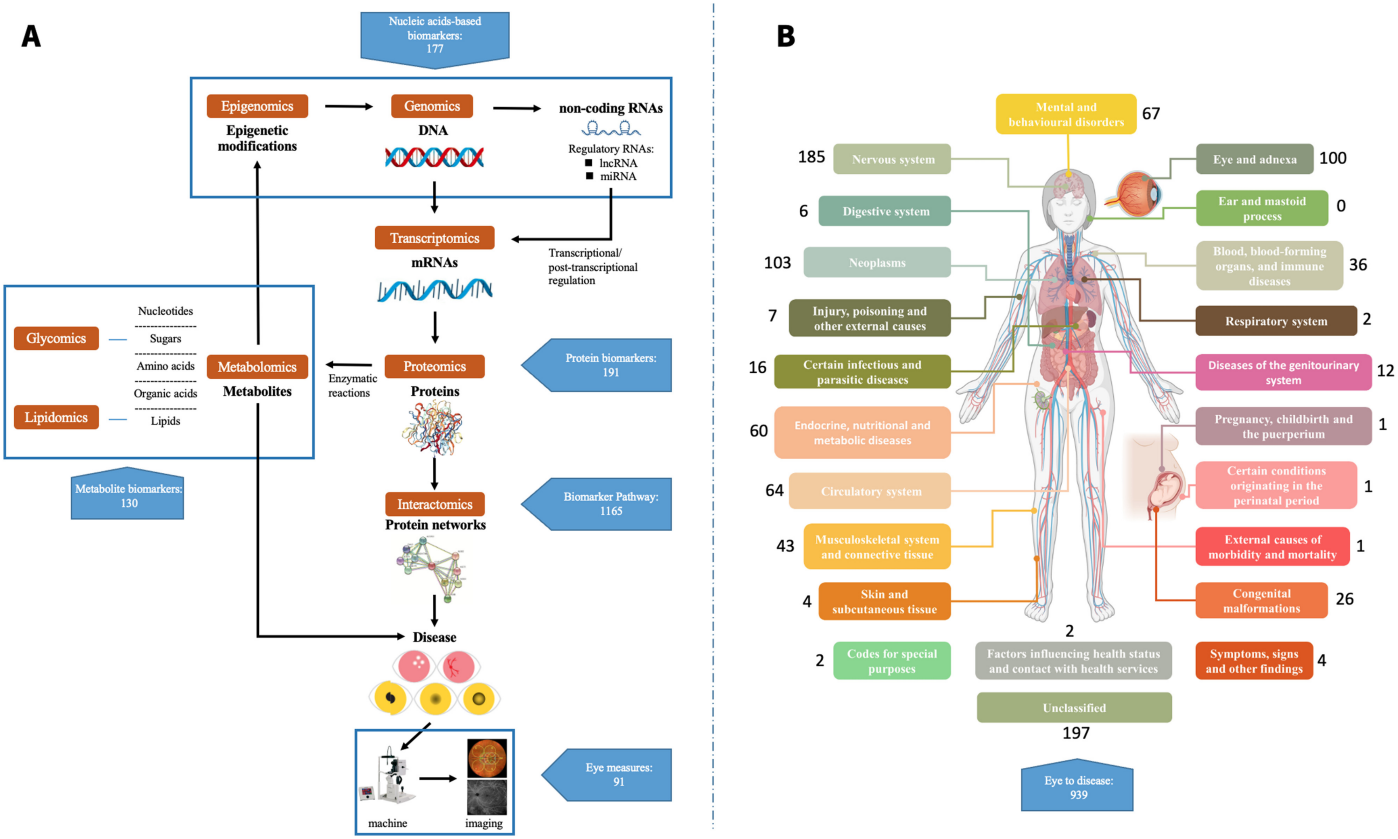
Data summary of the EBD. A. EBD includes 889 biomarkers for 26 eye diseases from 1196 studies. We classified these biomarkers according to their components, as 177 nucleic acids-based biomarkers (12 genetic locus biomarkers, 117 DNA, 35 miRNA, 8 mRNA, 1 DNA methylation, 3 lncRNA, and 1 circRNA), 191 protein biomarkers, and 130 metabolite biomarkers. Further, we also include 91 eye measures, 194 image biomarkers, and 106 other biomarkers (including cytokines, blood measures, diseases, symptoms, and therapies). B. For eye as biomarker for systemics diseases, EBD collects 939 eye biomarkers for 181 systemic diseases, from 890 studies.

The EBD contains 889 biomarkers for 26 eye diseases from 1196 studies containing 3 356 420 samples. We classified these biomarkers according to their components as 177 nucleic acids-based biomarkers (12 genetic locus biomarkers, 117 DNA, 35 miRNA, 8 mRNA, 1 DNA methylation, 8 mRNA, 3 lncRNA, 1 circRNA, 191 protein biomarkers, and 130 metabolite biomarkers). Further, we also included 91 eye measures and 194 image biomarkers. Meanwhile, 106 other biomarkers (including cytokines, blood measures, diseases, symptoms, and therapies) have been included in “Other biomarker” folder (Fig. 2A). For the eye itself as the biomarker, EBD has collected 939 uses of the eye as a biomarker for 181 systemic diseases, from 890 studies (Fig. 2B).

### 3.3 Functional category for biomarkers

The EBD includes 381 prediction biomarkers, 261 diagnosis biomarkers, 24 treatment biomarkers, and 131 prognosis biomarkers (Supplementary Fig. S1A). In this version of EBD, 78 nonbiomarkers—objects that have been proven not to have diagnostic or prognostic utility—have also been collected and stored.

### 3.4 BBI networks

**Figure 3:**
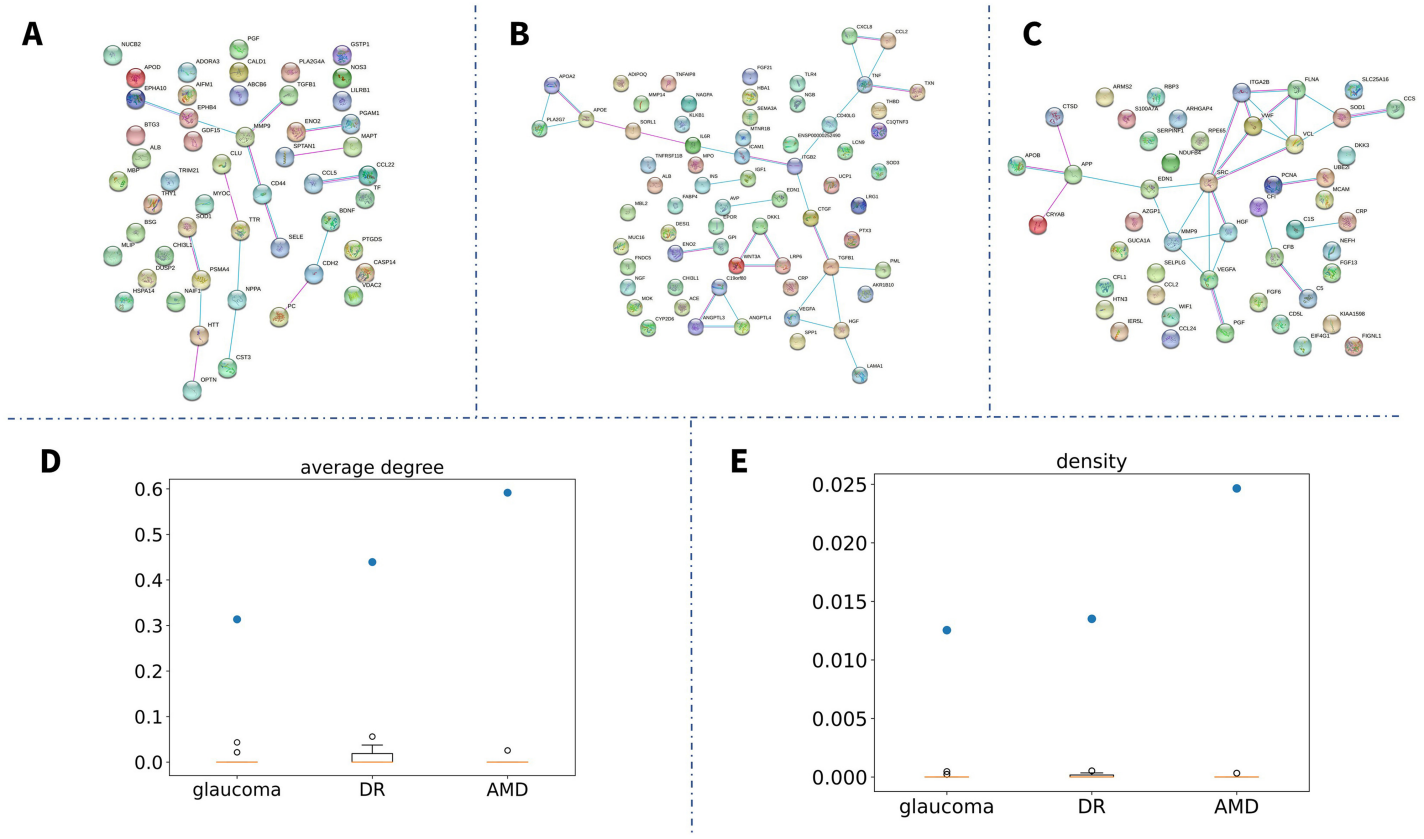
(A) BBI network of glaucoma; (B) BBI network of DR; (C) BBI network of AMD; (D) Boxplot for average degree of BBI networks (blue point) and random networks (yellow box); (E) Boxplot for density of BBI networks (blue point) and random networks (yellow box).

Glaucoma, DR, AMD, RE, and cataracts are the five most common eye diseases involving the most studied biomarkers (Supplementary Fig. S1B). Proteins are the most common biomarkers for eye diseases (Supplementary Fig. S1C). We extracted the protein biomarkers for these five common eye diseases separately and mapped them on the human PPI network to construct BBI networks for different eye diseases (Fig. 3). Since RE and cataract had too few protein biomarkers to construct networks, we only presented the BBI networks for glaucoma (Fig. 3A), DR (Fig. 3B), and AMD (Fig. 3C). We found that most BBI networks showed a low level of connectivity (low average degree and density; Fig. 3), indicating that most of the biomarkers were separated from others. In order to test, if the node in the String networks reported in this study was more than expected by chance, we randomly selected the same number of proteins from the 67 592 464 human protein list stored in the String database, to construct the same size networks with our BBI networks, and calculated their average degree and density. The comparison of these network topology features between our BBI networks and the randomly generated networks was shown in Fig. 3D and E, which indicated that our BBI networks showed much higher connectivity (higher average degree and density) than the random networks. Hence, we proved that the connectivity of BBI networks in this study was more than expected by chance.

### 3.5 Pathways for biomarkers

**Figure 4:**
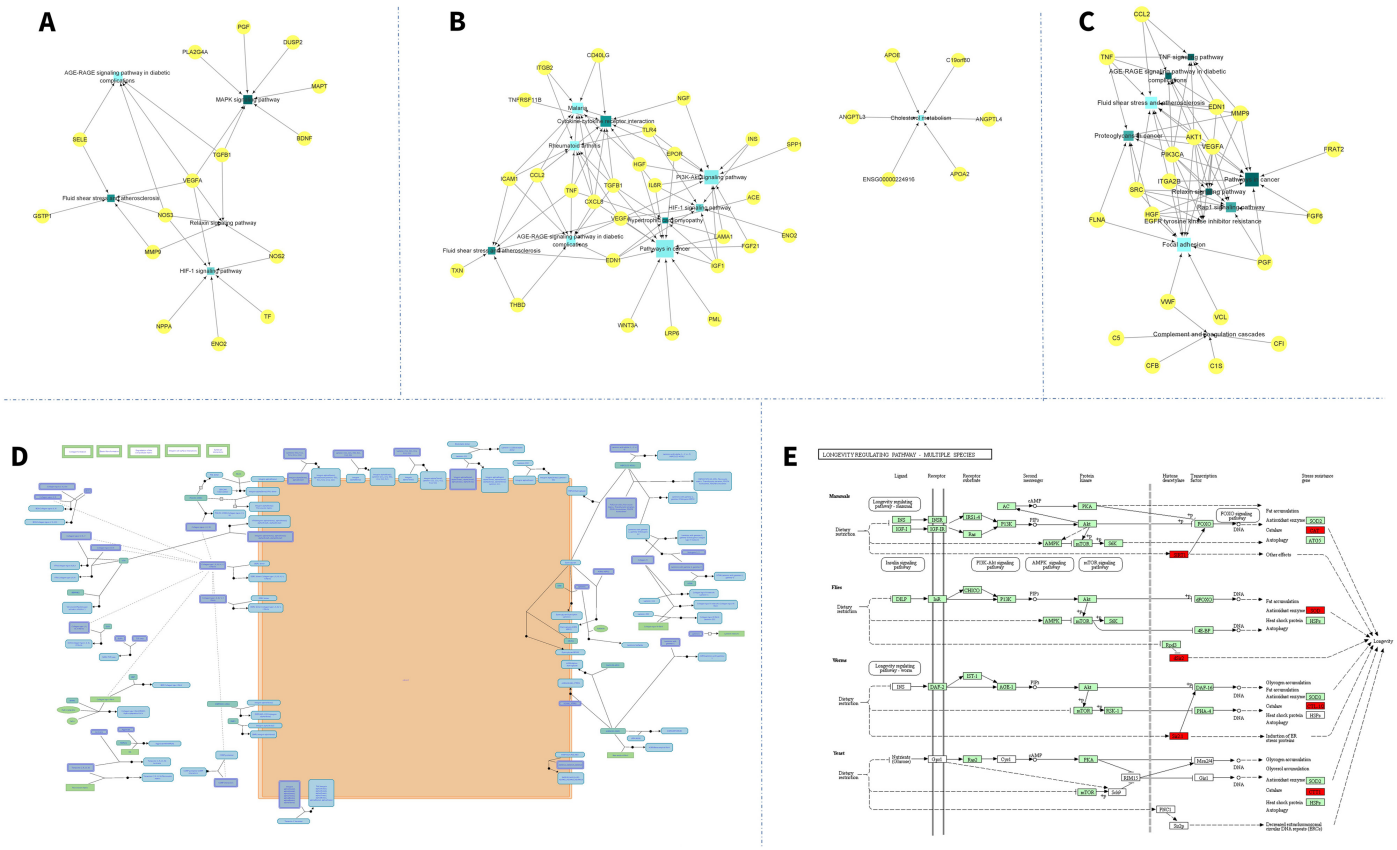
Biological pathways-biomarkers interaction networks for five major eye diseases. The yellow points meant the biomarkers, and they were connected by the enriched pathways, which were presented as blue points. (A) Glaucoma biomarkers were mapped on the HIF-1 signaling pathway, MAPK signaling pathway, Fluid shear stress and atherosclerosis, and AGE-RAGE signaling pathway in diabetic complications. (B) DR biomarkers were enriched on 57 pathways, and the Malaria, Rheumatoid arthritis, and AGE-RAGE signaling pathway in diabetic complications were the three most significant pathways. (C) For AMD, the Focal adhesion, Fluid shear stress and atherosclerosis, and Rap1 signaling pathway were the most significant pathways. (D) The Extracellular matrix organization was the only enriched pathway for RE. (E) In cataracts, the Longevity regulating pathway was the only significant pathway.

GO annotation and pathway enrichment analysis was conducted to find significant pathways for eye disease biomarkers (Supplementary Table S4), and we found that most of the biomarkers were mapped on several specific pathways. For glaucoma, biomarkers were mapped on five pathways (Fig. 4A), among which four were overlapped with AMD and DR: hypoxia-inducible factor 1 (HIF-1) signaling pathway, MAPK signaling pathway, Fluid shear stress and atherosclerosis, and AGE-RAGE signaling pathway in diabetic complications (Fig. 4A). For DR, 57 pathways were enriched (Supplementary Table S4), and the Malaria, Rheumatoid arthritis, and AGE-RAGE signaling pathways in diabetic complications were the three most significant pathways (Fig. 4B). For AMD, the Focal adhesion, Fluid shear stress and atherosclerosis, and Rap1 signaling pathway were the most significant pathways (Fig. 4C). We found 34 common pathways for DR and AMD biomarkers. The Extracellular matrix organization was the only enriched pathway for RE (Fig. 4D). In cataracts, the Longevity regulating pathway was the only significant pathway (Fig. 4E).

**Figure 5:**
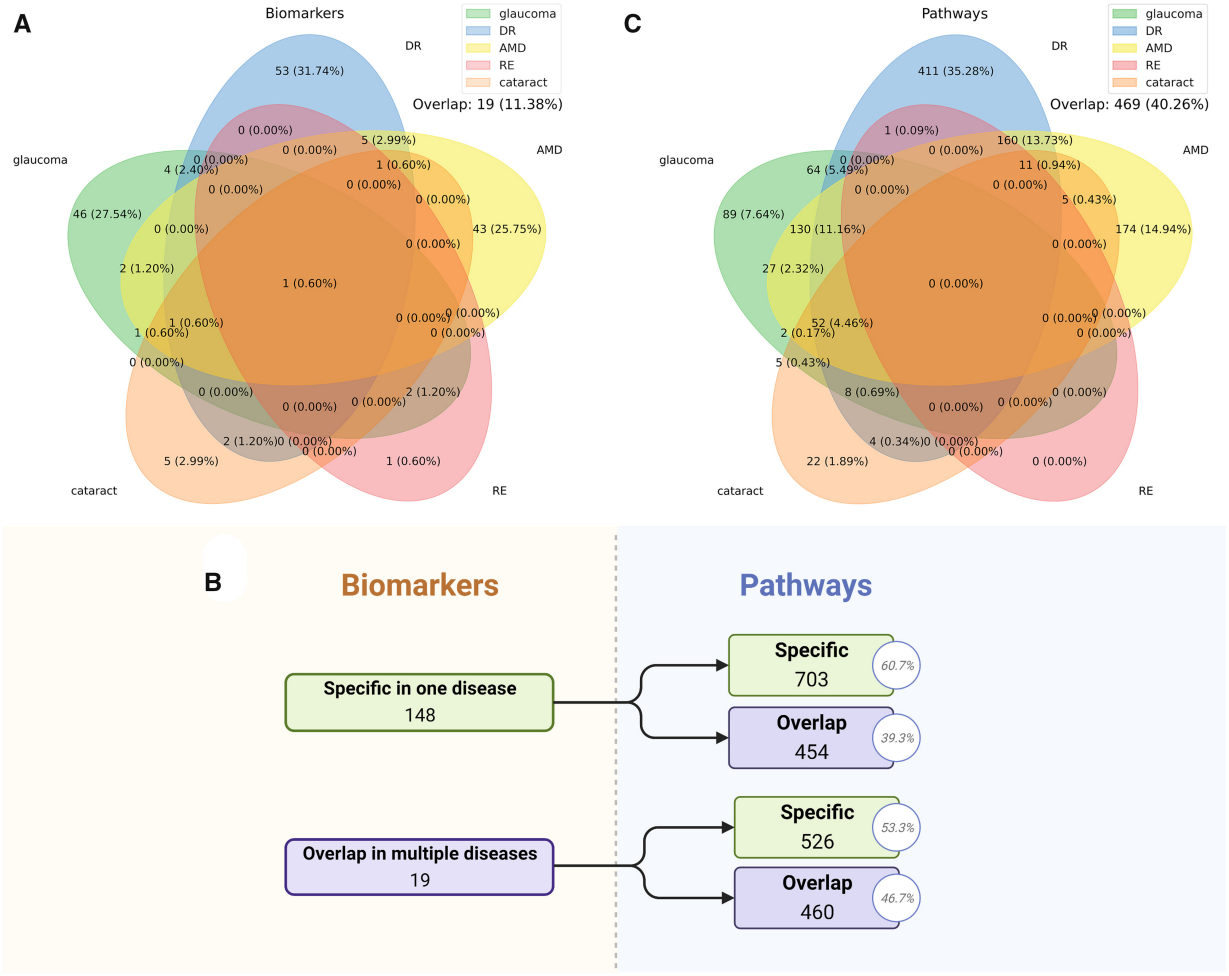
(A) Protein biomarker distribution in five major eye diseases. 19 biomarkers functioned in multiple diseases. (B) Distribution of biomarkers and pathways in specific or multiple diseases. (C) Pathway distribution in five major eye diseases. 469 pathways were enriched in multiple diseases.

In order to test the effect of randomizing biomarkers across disease classes on annotations, we first separated the protein biomarkers into two groups: specific functioned in one disease, or functioned in multiple diseases (Fig. 5A). We found that 926 pathways were enriched by multiple functional biomarkers, and 89, 174, 411, and 22 pathways were specifically enriched by biomarkers of glaucoma, AMD, DR, and cataract (Fig. 5B). No specific pathway was found for RE. Further, the bootstrap model showed that the mean false discovery rate (FDR) in enrichment is 0.01 (Table 1). We also calculated the enriched pathways that were specific in one disease or functioned in multiple diseases and found that 469 pathways (40.3% in total) functioned in multiple diseases. This evidence indicated that the links between a disease and a pathway do not survive randomization.

**Table 1.** Enrichment FDR estimated by the bootstrap method.

Disease	FDR (mean)	FDR (95% CI low)	FDR (95% CI high)
Glaucoma	0.0118	0.0104	0.0133
DR	0.0088	0.0080	0.0097
AMD	0.0109	0.0099	0.0121
RE	0.0147	NA	NA
Cataract	0.0138	0.0114	0.0165
All	0.0103	0.0098	0.0110

### 3.6 Biomarker expression

**Figure 6:**
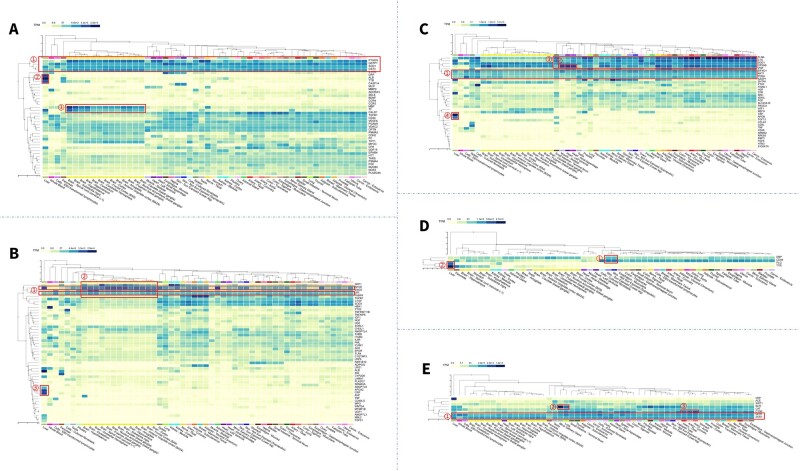
Biomarker expression in different tissues. (A) For glaucoma: PTGDS, GSTP1, SPD1, and CST3 expressed significantly high in almost all tissues; CRP, ALB, TTR showed markedly increase in liver; MBP and TF had high expression on brain. (B) For DR: GPI and APOE showed high expression in most of tissues; SPP1, APOE, ENO2, and GPI expressed high in the brain; ANGPTL8, APOA2, CRP showed high expression in the liver. (C) For AMD: EIF4G1, AKTI, and PCNA showed high stable expression in most tissues; CYAB showed obvious high expression in the heart and muscle; FLNA, C1S, SOD1, and CRYAB showed increased expression in the brain; CRP, APOB showed high expression in liver. (D) For RE: DBP and CD44 expressed high in the skin; PLG and TTR showed high expression in liver. (E) For cataracts: GPX1 and SOD1 expressed high in most of the tissues; SLP1 significantly expressed high in minor salivary gland and lung; CTGF expressed high in artery.

We also observed the expression of eye disease biomarkers on RNA-seq data (Fig. 6). We found that most of these biomarkers have stable expression levels among tissues, and several biomarkers showed significantly high expression in the liver and brain. For glaucoma, PTGDS, GSTP1, SPD1, and CST3 were expressed significantly higher in almost all tissues; CRP, ALB, and TTR were markedly increased in the liver; MBP and TF exhibited high expression in the brain (Fig. 6A). For DR, GPI, and APOE demonstrated high expression in most tissues (Fig. 6B). For AMD, EIF4G1, AKTI, and PCNA showed high expression in most tissues (Fig. 6C). For RE, DBP and CD44 expressed high in the skin, and PLG and TTR showed high expression in the liver (Fig. 6D). For cataracts, GPX1 and SOD1 were expressed highly in most tissues (Fig. 6E). We also mapped the biomarkers on scRNA-seq data (Supplementary Fig. S4).

## 4 Discussion

In this work, we presented a comprehensive platform of human eye biomarkers, EBD, encompassing 889 biomarkers for 26 eye diseases and 939 eye biomarkers for 181 systemic diseases. We collected biological/clinical biomarker information from 32 441 published papers, selected from 1881 original searching results, then curated them as the standard format and stored them in the EBD.

There are several biomarker databases for human complex diseases: CBD for colorectal cancers (Zhang et al. 2018), HFBD for Heart failure (He et al. 2021), and IDBD for infectious diseases (Yang et al. 2008). Our study fills the gap of a missing biomarker database for ophthalmology. Compared with previous biomarker databases, EBD first provided pathway enrichment and network function for protein biomarkers and added the biomarker pathways information. Meanwhile, EBD included some specific biomarkers like image biomarkers. Further, the display of nonbiomarkers could help researchers avoid prior mistakes and thus improve the precision of biomarker discovery.

Importantly, we mapped protein biomarkers to a human PPI network to construct BBI networks for five major eye diseases (Fig. 3). We found that these networks had high connections, indicating that biomarkers for eye diseases with high interplays. Combining different biomarkers as multiple biomarkers could increase the clinical effect significantly (Zhang et al. 2019). The appropriate selection of common biomarkers as a diagnostic and prognostic tool has so far remained elusive.

Additionally, we annotated protein biomarkers on 1165 biological pathways and stored them on EBD according to their corresponding diseases. We found that most of the biomarkers for eye diseases mapped on several specific pathways. Four common biomarker pathways were identified for glaucoma, AMD and DR (Supplementary Table S1). HIF-1 is a regulator for oxygen homeostasis, which is induced by oxygen availability, nitric oxide, and growth factors. The MAPK signaling pathway is a famous pathway for signaling from receptor to DNA. The Fluid shear stress and atherosclerosis play a master role in the progress of atherosclerosis. The binding of AGE to RAGE products NAPDH and enhances oxidative stress, plays an important role in the process of diabetic complications. This is the first systemic annotation in pathway level for eye diseases from biomarkers, which could further explain the mechanism of eye disease biomarkers and help future biomarker discovery.

We also conducted gene expression analysis for the five major eye diseases. We found some stably expressed biomarkers in most tissues, supporting their stability as effective biomarkers. Several biomarkers are expressed highly in the liver and brain, suggesting that they may be common biomarkers for both eye and relevant systemic disease, which may help inform a common pathophysiology.

We expect that the EBD resource will have a far-reaching impact on the identification of effective diagnostic and prognostic biomarkers for ocular and systemic disease. In particular, because EBD allows the end-user to simultaneously analyze any known biomarker for ocular and systemic diseases, it would greatly impact the prioritization of candidates from patient next-generation sequencing analysis, mapped intervals, and Genome-wide association studies (GWAS) studies. Indeed, future use of EBD can expedite the identification of new disease-linked genes, biomarkers, and drug targets. Future EBD updates will include adding new and wide range of biomarkers, providing more function such as actionable visualization and biomarker prediction, since in-depth clinical tie-ins to eye biomarkers have yet to be thoroughly performed. Meanwhile, the finding for eye diseases could be expanded to other complex diseases.

In summary, the conception of EBD provides a standardized platform for ocular biomarkers, and may be a future driver of ophthalmic precision medicine.

## Supplementary Material

btad194_Supplementary_Data

## Data Availability

The data underlying this article are available in the article and in its online supplementary material.

## References

[btad194-B1] Barabási AL. Scale-free networks: a decade and beyond. Science 2009;325:412–3. 19628854 10.1126/science.1173299

[btad194-B2] De las Rivas J , Fontanillo C. Protein-protein interaction networks: unraveling the wiring of molecular machines within the cell. Brief Funct Genomics 2012;11:48–96. 10.1093/bfgp/els036 22908212

[btad194-B3] Flaxman SR , Bourne RRA, Resnikoff S et al; Vision Loss Expert Group of the Global Burden of Disease Study. Global causes of blindness and distance vision impairment 1990–2020: a systematic review and meta-analysis. Lancet Glob Health 2017;5:e1221–34 29032195 10.1016/S2214-109X(17)30393-5

[btad194-B4] He H , Shi M, Lin Y et al HFBD: a biomarker knowledge database for heart failure heterogeneity and personalized applications. Bioinformatics 2021;37:4534–9. 34164644 10.1093/bioinformatics/btab470

[btad194-B5] London A , Benhar I, Schwartz M et al The retina as a window to the brain - from eye research to CNS disorders. Nat Rev Neurol 2013;9:44–53. 23165340 10.1038/nrneurol.2012.227

[btad194-B6] Strimbu K , Tavel JA. What are biomarkers? Curr Opin HIV AIDS 2010;5:463–6. 20978388 10.1097/COH.0b013e32833ed177 PMC3078627

[btad194-B7] Tamhane M , Cabrera-Ghayouri S, Abelian G et al Review of biomarkers in ocular matrices: challenges and opportunities. Pharm Res 2019;36:40. 30673862 10.1007/s11095-019-2569-8 PMC6344398

[btad194-B9] Tham Y-C , Li X, Wong TY et al Global prevalence of glaucoma and projections of glaucoma burden through 2040: a systematic review and meta-analysis. Ophthalmology 2014;121:2081–90. 24974815 10.1016/j.ophtha.2014.05.013

[btad194-B10] Vujosevic S , Parra MM, Hartnett ME et al Optical coherence tomography as retinal imaging biomarker of neuroinflammation/neurodegeneration in systemic disorders in adults and children. Eye (Lond) 2023;37:203–19. 35428871 10.1038/s41433-022-02056-9 PMC9012155

[btad194-B11] Wolf J , Boneva S, Schlecht A et al The human eye transcriptome atlas: a searchable comparative transcriptome database for healthy and diseased human eye tissue. Genomics 2022;114:110286. 35124170 10.1016/j.ygeno.2022.110286

[btad194-B12] Wong CW , Wong TY, Cheng C-Y et al Kidney and eye diseases: common risk factors, etiological mechanisms, and pathways. Kidney Int2014a;85:1290–32. 24336029 10.1038/ki.2013.491

[btad194-B13] Wong TY , Rosamond W, Chang PP et al Retinopathy and risk of congestive heart failure. JAMA 2005;293:63–9. 15632337 10.1001/jama.293.1.63

[btad194-B14] Wong WL , Su X, Li X et al Global prevalence of age-related macular degeneration and disease burden projection for 2020 and 2040: a systematic review and meta-analysis. Lancet Glob Health2014b;2:e106–16. 25104651 10.1016/S2214-109X(13)70145-1

[btad194-B15] World Health Organization. World Report on Vision. Geneva*:* World Health Organization, 2019.

[btad194-B16] Yang IS , Ryu C, Cho KJ et al IDBD: infectious disease biomarker database. Nucleic Acids Res 2008;36:D455–60. 17982173 10.1093/nar/gkm925 PMC2238845

[btad194-B17] Yuan J , Chen F, Fan D et al EyeDiseases: an integrated resource for dedicating to genetic variants, gene expression and epigenetic factors of human eye diseases. NAR Genom Bioinform 2021;3:iqab050. 10.1093/nargab/lqab050 PMC8168129 34085038

[btad194-B18] Zhang X , Sun X-F, Cao Y et al CBD: a biomarker database for colorectal cancer. Database 2018;2018:bay046 29846545 10.1093/database/bay046 PMC6007224

[btad194-B19] Zhang X , Sun X-F, Shen B et al Potential applications of DNA, RNA and protein biomarkers in diagnosis, therapy and prognosis for colorectal cancer: a study from databases to AI-assisted verification. Cancers (Basel) 2019;11:172. 30717315 10.3390/cancers11020172 PMC6407036

[btad194-B20] Zhu Z , Shi D, Guankai P et al Retinal age gap as a predictive biomarker for mortality risk. Br J Ophthalmol 2023;107:547–54. 35042683 10.1136/bjophthalmol-2021-319807

