# Supplementary figures and images for: EBD: an eye biomarker database
Source: Bioinformatics. 2023 Apr 13;39(5):btad194. doi: 10.1093/bioinformatics/btad194 (PMC10168589; doi:10.1093/bioinformatics/btad194)

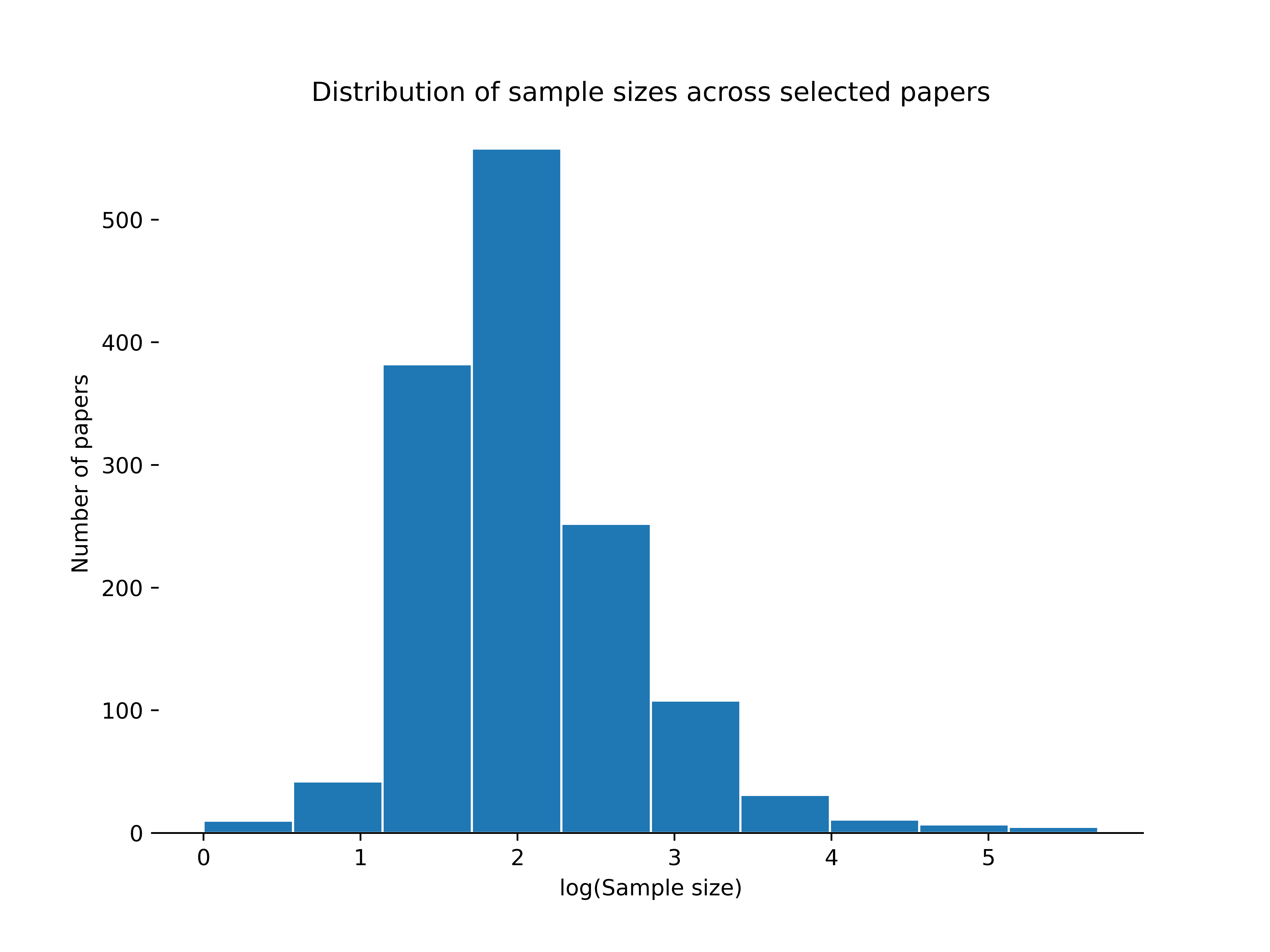

Supplement: btad194_Supplementary_Data [file btad194_supplementary_data.zip › Figure S2.png]

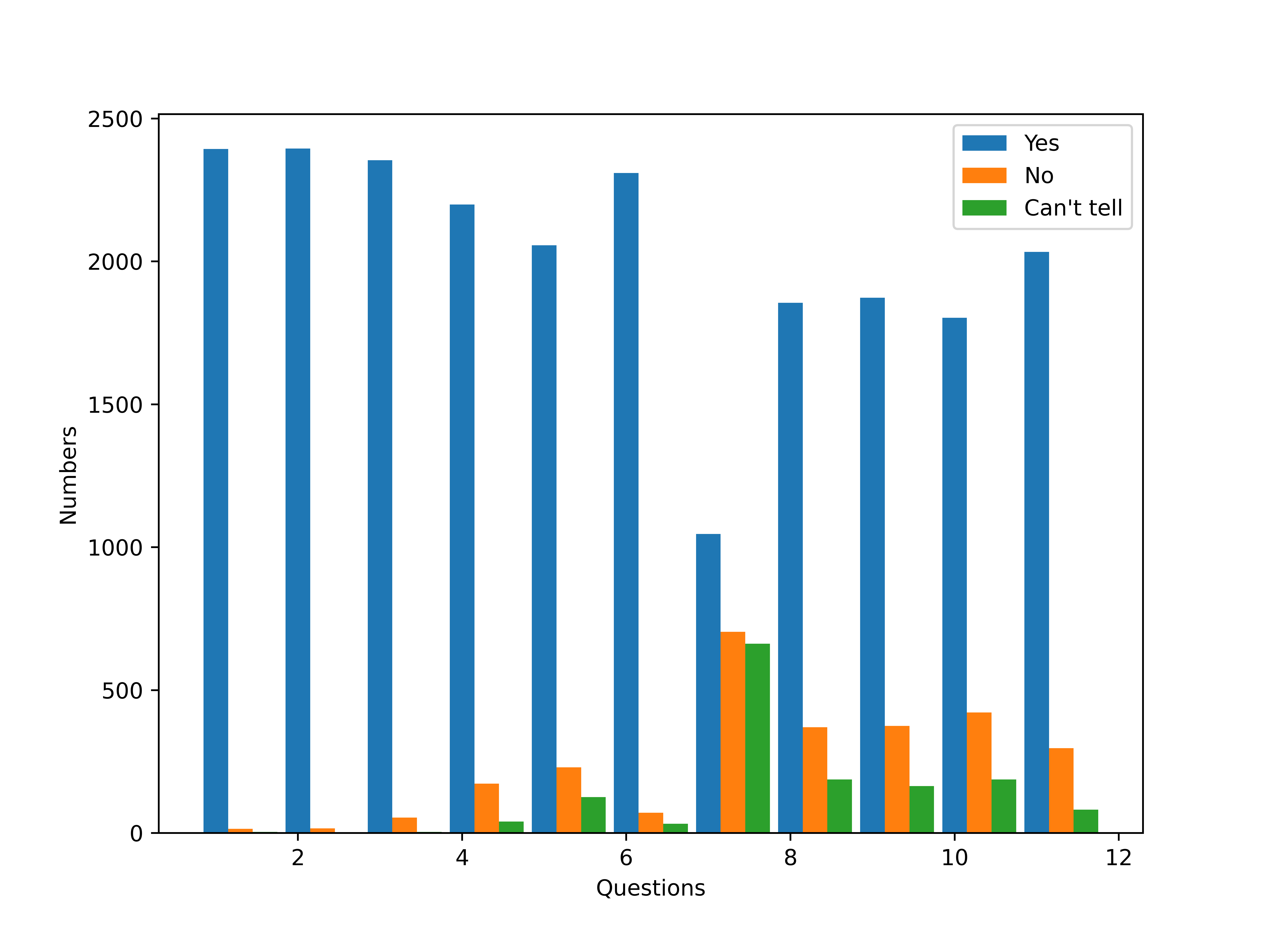

Supplement: btad194_Supplementary_Data [file btad194_supplementary_data.zip › Figure S3.png]

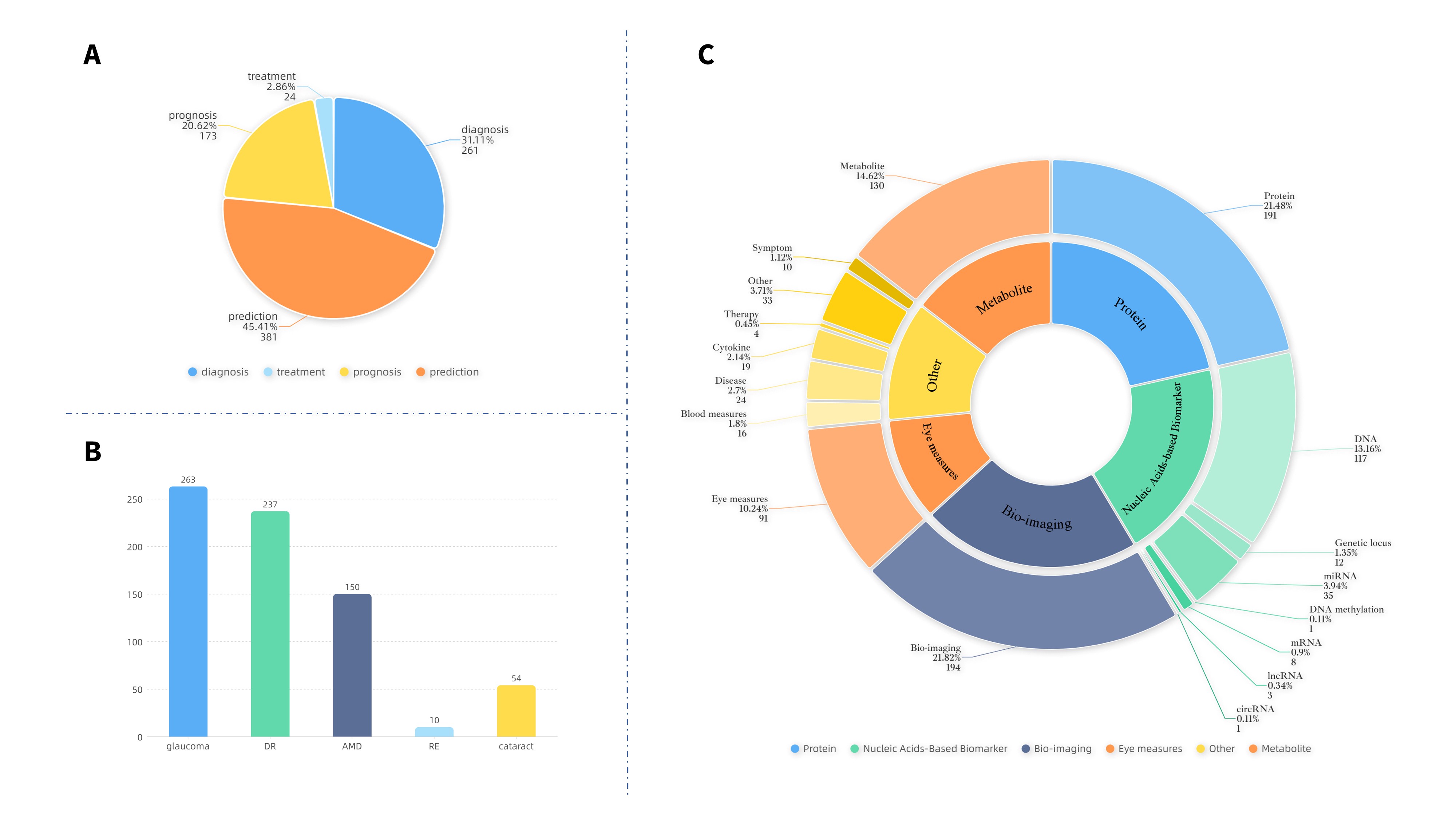

Supplement: btad194_Supplementary_Data [file btad194_supplementary_data.zip › Figure S1.jpeg]
